# Knowledge, Attitude, and Practice toward Skin Cancer Prevention and Detection among Jordanian Medical Students: A Cross-Sectional Study

**DOI:** 10.1155/2022/6989827

**Published:** 2022-02-14

**Authors:** Khaled Seetan, Almu'atasim Khamees, Afnan Migdadi, Mosab Abu Shqeer, Maram Jameel Hasan, Leen Ahmad Shatnawi, Tala Abu Bakr, Nada Zayed

**Affiliations:** ^1^Department of Clinical Sciences, Faculty of Medicine, Yarmouk University, Irbid, Jordan; ^2^Faculty of Medicine, Hashemite University, Zarqa, Jordan; ^3^Department of Clinical Dermatology, Lincoln County Hospital, Lincoln, UK

## Abstract

**Introduction:**

Skin cancer is one of the most growing types of cancer, especially in the Mediterranean, even though it is a preventable disease. The purpose of this study is to assess medical students' knowledge, attitude, and practice about skin cancer prevention and detection.

**Methods:**

A cross-sectional study was conducted using a validated structured questionnaire covering the areas of knowledge, attitude, and practice of the study participants.

**Results:**

The study involved 1530 students; 55.3% were females. Most of the students possessed proper knowledge about skin cancer (81%). The most prevalent skin cancer risk factors were sun exposure during the day (83.5%) and immunosuppression (71.2%). More than half of the students did not have any habits of skin examination (61.5%). 20% of the students never used sunscreen, while only 20% of them avoided sun exposure during day hours.

**Conclusion:**

The general level of the medical students' knowledge of skin cancer and its risk factors appeared to be higher than what is found in other studies; it is reasonable as the study participants were medical students. However, the protective behavior from the sun was inadequate when compared to the level of knowledge reported. Additional education about the behavior toward sun exposure and protection against skin cancer may be needed to be implemented in the dermatology curriculum.

## 1. Introduction

Skin cancer is the most common cancer in the United States, with an estimated lifetime risk of 1 in every two people [1, 2]. The two most common types of skin cancer are basal cell carcinoma and squamous cell carcinoma, respectively, while malignant melanoma is less common, with an incidence of 1 in every 40 [3, 4]. However, despite the lower prevalence, malignant melanoma remains the most common cause of skin-related cancer [3, 5]. In the United States, it is estimated that skin cancer's average annual economic cost is $8.1 billion, with a dramatic rise in the total cost compared with other cancers [6].

Research has shown that the average annual cost of skin cancer from 2002–2006 to 2007–2011 increased by 126.2%, and the average annual cost of all other cancers increased by 25.1% [6]. Hence, the importance of interventions and strategies that aim to reduce the health and economic burden of skin cancer. One such strategy is improving patient health education that promotes sun-protective behaviors. Primary sun-protective behaviors involve the reduction of natural and artificial UV light exposure, the application of sunscreen with a good sun protection factor (SPF), wearing hats with adequate protection to the entire head, along with tightly woven clothing that covers the arms, torso, and legs [7]. At the same time, secondary sun-protective behaviors include the regular conduct of self-skin and total body skin examinations by physicians [4]. Reports suggest that avoidance of exposure to natural and artificial UV light could prevent more than 5 million cases of skin cancer yearly [8], and daily application of a sunscreen with SPF 15 or more could decrease the risk of squamous cell carcinoma by 40% and melanoma by 50% [9].

Researchers worldwide have continuously tried to address the problem behind the rising incidence of skin cancer, and many environmental and genetic causes have been discovered [10].

A study with nursing students revealed that only 8% of students performed the sun-protection recommendations, and this was the least correctly performed activity [11]. Accordingly, training the students in the medical-related fields, mainly those directed towards primary care, about the importance of sun-protective behaviors is crucial to decreasing skin cancer incidence. Primary care physicians possess more chances to conduct skin examinations and educate patients on sun-protective behaviors [12]; recent reports pointed out the importance of health education of medical students about the effect of sun exposure on human health [13–16].

The purpose of this study is to assess medical students' knowledge, attitude, and practice about skin cancer prevention and detection.

## 2. Methodology

This descriptive cross-sectional survey-based study was conducted in Jordanian medical schools between July and October 2021. The survey was conducted using a validated structured questionnaire developed by researchers with help from the literature review of similar published studies to cover critical areas. Moreover, the survey was validated by three experts from the departments of dermatology, general surgery, and public health at the same affiliated university, Yarmouk University, Faculty of Medicine. We have included the questionnaire in the revised manuscript.

The questionnaire consisted of 51 questions categorized to cover all survey targets. The first six questions were about the student's demographic data; the following 19 questions were meant to test the knowledge of the students about the risk factors of skin cancer in general, adding 26 questions about the impact of sun exposure on the skin and the behaviors of participants toward sun protection and early skin cancer signs. Participation in the study was voluntary, and consent was taken from each participant after explaining the purpose of the research. All medical students from all Jordanian medical schools who gave consent to participate in this study were included. The study was approved by the institutional board review at Yarmouk University, number RD/119/12/1870.

A structured online Google form was created to aid in data collection as a study tool. The online survey link was distributed through social media platforms, through universities' representatives, and universities' official emails. After that, data were collected and organized into an Excel document then imported to The Statistical Package for Social Sciences (SPSS) version 25, where coding and data analysis were performed. Descriptive statistical methods were used to evaluate the data using mean ± SD for continuous variables and frequencies and percentages for categorical variables. Inferential statistics in the form of a Chi-Square test were done to evaluate the significance of the association between participants' gender and knowledge about skin cancer. *P* values of less than 0.05 were considered to be statistically significant.

## 3. Results

A total of 1530 subjects were recruited for the study, representing around 10% of the entire student population at the time of the study. Females constituted 55.3% of the study participants (846), while 684 males were selected. The majority of the study population was found within the age group 20–23 (67.5%). The study involved participants from the first year to the sixth year, with the fifth-year students representing the majority (28.1%). Most of the students (44.4%) scored very good degrees as their average academic rank, followed by excellent (32%), good (22.5), and weak (1%).  Table 1.

Regarding their knowledge about skin cancer, most of the students have encountered a family history of skin cancer (97.9%), and the majority of them considered themselves to possess proper knowledge (81%). At the same time, the same percentage selected medical school as their source of information regarding skin cancer. About 46.9% of the participants correctly identified basal cell carcinoma as the most common type of skin cancer. The majority of them identified melanoma characteristics as a mole with an asymmetrical border (63%), while more than half of the students selected it as changes in color (59.9%).  Table 2.

When testing the general knowledge about the risk factors of the disease, most of the participants (83.5%) acknowledged going out during the hours of 10 am to 4 pm without sun protection as a risk factor for the disease. More than half (58.7) considered the effect of smoking and alcohol in increasing the risk. Having fair skin was a risk in the opinion of 64%, and exposure to ultraviolet radiation was identified by 95.2% of the participants as a risk. More than half (59.5%, 57.8%, and 57.4%) considered having lots of nevi, freckles, and moles in the body, tanning, and acquiring painful sunburn before 20 years old to be risk factors, respectively. Immunosuppressive agents were a risk in the responses of 71.2%, while those not using sunscreens were correctly identified by 63%. Other responses to general knowledge and risk factors are shown in Table 3.

Regarding the attitude toward the disease, most of the study subjects (61.5%) claimed that they did not have any habit of doing a self-skin examination. Most of those who did not perform a self-skin examination (91%) had never even thought of it, among the others who usually perform self-skin examinations. Most of them do the exam to look for new lesions (72%), and early skin cancer detection (53.7%). Half of the participants learned about skin self-examination from medical courses and lectures; others heard it from social media (28.9%) and dermatologists (19.4%). The rest of the attitude responses are shown in Table 4.

Only 20.3% stated that they always avoid the sun from 10 am to 4 pm, while only 9.4% and 19.1% wear hats or sunglasses when going out, respectively. Other responses to practice questions are demonstrated in Table 5.

No statistically significant association between study variables has been found.

## 4. Discussion

Skin cancer is among the most prevalent skin cancers worldwide and is a growing health concern in the Mediterranean region. Generally, cancers constitute the second most common cause of death, outnumbered only by cardiovascular diseases [17]. Since skin cancer can be prevented, given that many risk factors have been identified, the WHO has implemented strategies to control skin cancer by raising people's knowledge about skin cancer through health education [17], improving attitudes and performance. However, Romero-Collado A et al., who conducted their study among primary care nurses in Spain, found that only 11 out of 137 nursing students (8.0%) performed sun protection recommendations. This was the least activity performed and reflects poor skills in health prevention according to skin cancer [11]. This highlights an additional point about the importance of health education about skin cancer: it is reasonable to work on improving primary and secondary prevention rather than the management of the disease, which, as an example, costs more than 400 million dollars every year in Australia [18].

The statistical analysis showed that the level of knowledge about skin cancer and its risk factors is generally reasonable. Nearly half of our participants correctly identified basal cell carcinoma as the most common type of skin cancer. A similar study among medical students showed a better percentage (67.8%) [19]. Third of the participants correctly disagreed about the fact that tanning salons offer a safer alternative to tanning outdoor, although less than what is found by Ivanov (98.3%), while only 20% agreed that having many moles increased the risk of having skin cancer, again the percentage is high compared to the American study (71.9%) [19].

Sun exposure is a risk factor for skin cancer, especially in a region with rich sunlight like Jordan. The participants in the current study showed a considerable variability when asked about the risk factors that could lead to the occurrence of skin cancer, and most of them agreed that genetic factors play a role (93.3%), a higher level than the study conducted in Italy (44.3%). However, it is worth mentioning that it was done in secondary school rather than medical students, where proper knowledge about the disease might have already been conducted [20]. In addition, sun exposure during the day with high sunrays within protection was also a popular risk factor (83.5%), slightly higher than a study done in Saudi Arabia where 77% agreed [21].

More than half of the students stated that they did not have the habit of doing skin examinations, which is an unsatisfactory result given that the population under study was medical students. A similar issue was found among nurse practitioners, as only 22% feel confident in performing skin examinations on their patients, and 67% of them feel less confident in counseling their patients about it [10].

Regarding the practice of our study participants, it was average and unsatisfactory at some points. It is well known that ultraviolet radiation, particularly that of the wavelength 290–320 nm, is a risk factor for skin cancer due to its DNA-damaging and immunosuppressive effects [22]. Although the association between UV light exposure and skin cancer was highly reported by our study participants (95.2%), the results showed that only 20% of the students respected the time restrictions and avoided the sun during the day where maximum radiation levels are present.

Although 39.3% of them always use sunscreen, which is surprisingly lower compared to a similar study by MahmoodAbad, as 60% of the students under the study used sunscreen and lower than the 73% found in the Martin study and Suppa (78.7%) [20, 23]. Although a higher level of practice was identified, there was still an area of ignorance toward sun exposure as a risk factor. In addition, the same percentage was found in a similar study among medical students in the USA [19].

As sun exposure is one of the risk factors of skin cancer, wearing long pants and long-sleeved clothes can be protective; this method of protection was utilized by 47.3% of the students, which is higher than the Italian study where only 7% of the participants used protective clothes [20].

In our study, the general knowledge and attitude toward skin cancer were generally good, given that the participants were medical students; their level of knowledge about the disease may already be good, and the sources of information were the most reliable ones. Conversely, the results of a similar study conducted among other students from different fields, including medical students, showed that the level of knowledge and attitude among the nonmedical students was lower than that of medical students [17].

## 5. Conclusion

The general level of the medical students' knowledge of skin cancer and its risk factors appeared to be higher than what is found in other studies; it is reasonable as the study participants were medical students. However, the protective behavior from the sun was inadequate when compared to the level of knowledge reported. Additional education about the behavior toward sun exposure and protection against skin cancer may be needed to be implemented in their dermatology curriculum.

### 5.1. Limitations

The main limitation of this study is using an online questionnaire, which can lead to the possibility of self-reporting bias. However, we avoid this problem by using a large representative sample of medical students.

## Figures and Tables

**Table 1:** Demographic and general information about study participants.

		*N*	*N* (%)
Age groups in years	Less than 20	126	8.2
	20–23	1032	67.5
	24–27	368	24.1
	28 or more	4	0.3
Gender	Female	846	55.3
	Male	684	44.7
Study year	First-year	82	5.4
	Second-year	172	11.2
	Third-year	174	11.4
	Fourth-year	370	24.2
	Fifth-year	430	28.1
	Sixth-year	302	19.7

Average academic rank	Weak	16	1.0
	Good	344	22.5
	Very good	680	44.4
	Excellent	490	32.0

Do you have a family history of skin cancer	No	1498	97.9
	Yes	32	2.1

**Table 2:** General knowledge of participants about skin cancer mainly melanoma.

		Gender						*p* (*X*^2^)
		Female		Male		Total		
		*N*	*N* (%)	*N*	*N* (%)	*N*	*N* (%)	
Do you have knowledge about skin cancer	No	138	16.3	152	22.2	290	19.0	0.003^*∗*^ (8.6)
	Yes	708	83.7	532	77.8	1240	81.0	
What is your major source of information about skin cancer	Books	186	24.0	230	36.9	416	29.8	<0.001^*∗*^ (38.9)
	Medical school	626	80.9	506	81.1	1132	81.0	
	Course outside medical school	36	4.7	36	5.8	72	5.2	
	Health professional	120	15.5	108	17.3	228	16.3	
	Friend/relative	31	4.0	33	5.3	64	4.6	
	Internet	294	38.0	264	42.3	558	39.9	
	Media	150	19.4	90	14.4	240	17.2	
According to your knowledge, what is the most common form of skin cancer?	Angiosarcoma	2	0.2	12	1.8	14	0.9	0.002^*∗*^ (18.9)
	Basal cell carcinoma	408	48.2	310	45.3	718	46.9	
	Melanoma	148	17.5	114	16.7	262	17.1	
	Squamous cell carcinoma	158	18.7	160	23.4	318	20.8	
	T Cell lymphoma	10	1.2	2	0.3	12	0.8	
	Don't know	120	14.2	86	12.6	206	13.5	

How many moles (melanocytic nevi) does the average person have?	5	88	23.5	60	23.6	148	23.6	0.003^*∗*^ (16.1)
	10	88	23.5	90	35.4	178	28.3	
	25	142	38.0	76	29.9	218	34.7	
	50	50	13.4	20	7.9	70	11.1	
	100	6	1.6	8	3.1	14	2.2	

What is the most common melanoma location?	Head	146	17.3	126	18.4	272	17.8	0.025^*∗*^ (14.4)
	Palm and sole	42	5.0	44	6.4	86	5.6	
	Trunk	201	23.8	140	20.5	341	22.3	
	Legs	60	7.1	34	5.0	94	6.1	
	Mucosa	22	2.6	34	5.0	56	3.7	
	Arms	84	9.9	84	12.3	168	11.0	
	Don't know	290	34.3	222	32.5	512	33.5	

What are the melanoma characteristics	Change of color/several colors	554	65.5	362	52.9	916	59.9	<0.001^*∗*^ (44.8)
	Wound that does not heal	128	15.1	74	10.8	202	13.2	
	Mole with asymmetric borders	562	66.4	402	58.8	964	63.0	
	Pigmented lesion	552	65.2	420	61.4	972	63.5	
	Don't know	154	18.2	144	21.1	298	19.5	

**Table 3:** General knowledge about risk factors for skin cancer.

	Disagree		Don't know/neutral		Agree	
	*N*	%	*N*	%	*N*	%
Going out during the hours of 10.00 am to 4.00 pm without sun protection	98	6.4	154	10.1	1278	83.5
Smoking/alcohol use	328	21.4	304	19.9	898	58.7
Having fair skin	224	14.6	318	20.8	988	64.6
Exposure to ultraviolet radiation	38	2.5	36	2.4	1456	95.2
Lots of nevus, freckles, and moles on the body	294	19.2	326	21.3	910	59.5
Having never tanning skin type	604	39.5	526	34.4	400	26.1
Solarium/tanning salons	208	13.6	438	28.6	884	57.8
Having light-colored eyes	626	40.9	424	27.7	480	31.4
Having red or blonde hair	614	40.1	418	27.3	498	32.5
Having sun burns which are painful and bubbly before 20 years of age	300	19.6	352	23.0	878	57.4
Exposure to petroleum, coal, or arsenic	162	10.6	302	19.7	1066	69.7
Family history of skin cancer	58	3.8	44	2.9	1428	93.3
Taking immunosuppressive treatment	150	9.8	290	19.0	1090	71.2
Not using sunscreen	174	11.4	392	25.6	964	63.0
You are adequately protected from UV rays with thin cloud cover	588	38.4	362	23.7	580	37.9
Tanning salons offer a safe alternative to sun tanning outdoors	598	39.1	598	39.1	334	21.8
People with many moles are at an increased risk of developing melanoma	292	19.1	300	19.6	938	61.3
Wet clothing offers less protection against the sun than dry clothing	452	29.5	746	48.8	332	21.7
When you are swimming in a pool, the part underwater is protected from the sun since water reflects most of the UV light	426	27.8	492	32.2	612	40.0
Using self-tanning lotions or cream is an effective method for sun protection	542	35.4	390	25.5	598	39.1
The ozone layer filters most of the ultraviolet type B but little of the A type	144	9.4	734	48.0	652	42.6
A suntan offers adequate protection to prevent sunburn	556	36.3	524	34.2	450	29.4
Chemical sunscreens give optimal protection as soon as they contact the skin	534	34.9	396	25.9	600	39.2
Sunblock SPF mean (sun protective factor)	82	5.4	430	28.1	1018	66.5

**Table 4:** Attitudes toward skin self-examination.

		*N*	%
Your attitude toward skin protective behaviors and self-skin exam: do you have the habit of doing the skin self-examination?	No	942	
	Yes	588	

If not, why?	I have learned about it but being scared of finding something	46	
	It is not necessary	38	
	Never thought about it	858	

What do you look for in the skin self-examination	Changing mole	406	69.0
	New mole	350	59.5
	Nodule	272	46.3
	New lesion	426	72.4

Reasons for performing skin self-examination	Early detection of skin cancer	316	53.7
	Fear of skin cancer	184	31.3
	Friend/relative had skin cancer	68	11.6
	Peace of mind	290	49.3
	Recommendation from doctor	130	22.1

Where did you learn about skin self-examination	Books	88	15.0
	Dermatologist/health care professional	114	19.4
	Friends/relatives	42	7.1
	Medical courses and lectures	294	50.0
	Internet	210	35.7
	Social media	170	28.9

**Table 5:** Practices of students toward sun exposure.

	Never		Rarely		Sometimes		Often		Always	
	*N*	%	*N*	%	*N*	%	*N*	%	*N*	%
I avoid the sun during the hours of 10 am to 4 pm	44	2.9	122	8.0	436	28.5	618	40.4	310	20.3
When I go out, I wear a hat	476	31.1	380	24.8	304	19.9	226	14.8	144	9.4
I wear sunglasses	322	21.0	330	21.6	352	23.0	234	15.3	292	19.1
When I go out, I use sunscreen at least with an SPF of 15	324	21.2	182	11.9	200	13.1	222	14.5	602	39.3
Wear sunscreen at least with an SPF of 15 while at the beach or swimming	310	20.3	166	10.8	238	15.6	218	14.2	598	39.1
I do not go to swim	204	13.3	202	13.2	386	25.2	338	22.1	400	26.1
I drink water minimum 8–10 glasses	18	1.2	124	8.1	336	22.0	428	28.0	624	40.8
I wear long pants and long-sleeved shirt at summer time	94	6.1	136	8.9	302	19.7	274	17.9	724	47.3
I go for body tanning regularly	816	53.3	266	17.4	200	13.1	108	7.1	140	9.2

## Data Availability

The data used to support the findings of this study are available from the corresponding author upon request.
